# Supplementary material for: Catalytic flexibility of rice glycosyltransferase OsUGT91C1 for the production of palatable steviol glycosides
Source: Nat Commun. 2021 Dec 2;12:7030. doi: 10.1038/s41467-021-27144-4 (PMC8639739; doi:10.1038/s41467-021-27144-4)
Supplement: Supplementary file 1 — Supplementary Information [file 41467_2021_27144_MOESM1_ESM.pdf]

Supplementary Information

Catalytic flexibility of rice glycosyltransferase OsUGT91C1 for the production of palatable steviol glycosides

Jinzhu Zhang[1]†, Minghai Tang[1]†, Yujie Chen[1]†, Dan Ke[1], Jie Zhou[1], Xinyu Xu[1], Wenxian Yang[1], Jianxiong He[1], Haohao Dong[1], Yuquan Wei[1], James H. Naismith[1,2,3], Yi Lin[4], Xiaofeng Zhu[1]*, Wei Cheng[1]*

Affiliations:

1 Key Laboratory of Bio-Resource and Eco-Environment of Ministry of Education, State Key Laboratory of Biotherapy and Cancer Center, West China Hospital of Sichuan University, College of Life Sciences, Sichuan University, Chengdu 610065, China

2 Division of Structural Biology, Wellcome Trust Centre of Human Genomics, Roosevelt Drive, Oxford OX3 7BN, UK

3 Rosalind Franklin Institute, Didcot, Oxon OX11 0FA, UK

4 International Peace Maternity and Child Health Hospital, Institute of Embryo-Fetal Original Adult Disease, School of Medicine, Shanghai Jiao Tong University, Shanghai 200030, China

† These authors contributed equally: Jinzhu Zhang, Minghai Tang, Yujie Chen

***These authors jointly supervised this work: Xiaofeng Zhu (zhuxiaofeng@scu.edu.cn), and Wei Cheng (chengwei669@scu.edu.cn)**

Supplementary Fig. 1 Reb A reaction with OsUGT91C1

Supplementary Table 1 Data collection and refinement statistics of X-ray structures

Supplementary Fig. 2 Experimental Fo-Fc difference electron density and the final refined 2Fo-Fc electron density for the bound ligands

Supplementary Fig. 3 Conformational change upon complex formation

Supplementary Fig. 4 Identical UDP binding in all complexes of OsUGT91C1

Supplementary Fig. 5 Role of His27 and Glu283 of OsUGT91C1 in β (1-2) sugar transfer

Supplementary Fig. 6 Comparison of the steviol compounds with the R2 end at the active site

Supplementary Fig. 7 Cleavage of β (1-2) glycosidic bond by OsUGT91C1

Supplementary Fig. 8 Role of His27 of OsUGT91C1 in β (1-6) sugar transfer

Supplementary Fig. 9 Engineering to enhance β (1-2) sugar transfer

Supplementary Fig. 10 Engineering to suppress the unwanted β (1-6) sugar transfer

Supplementary Table 2 Synthetic encoding sequence of OsUGT91C1, the primers, and the reagents used in this study

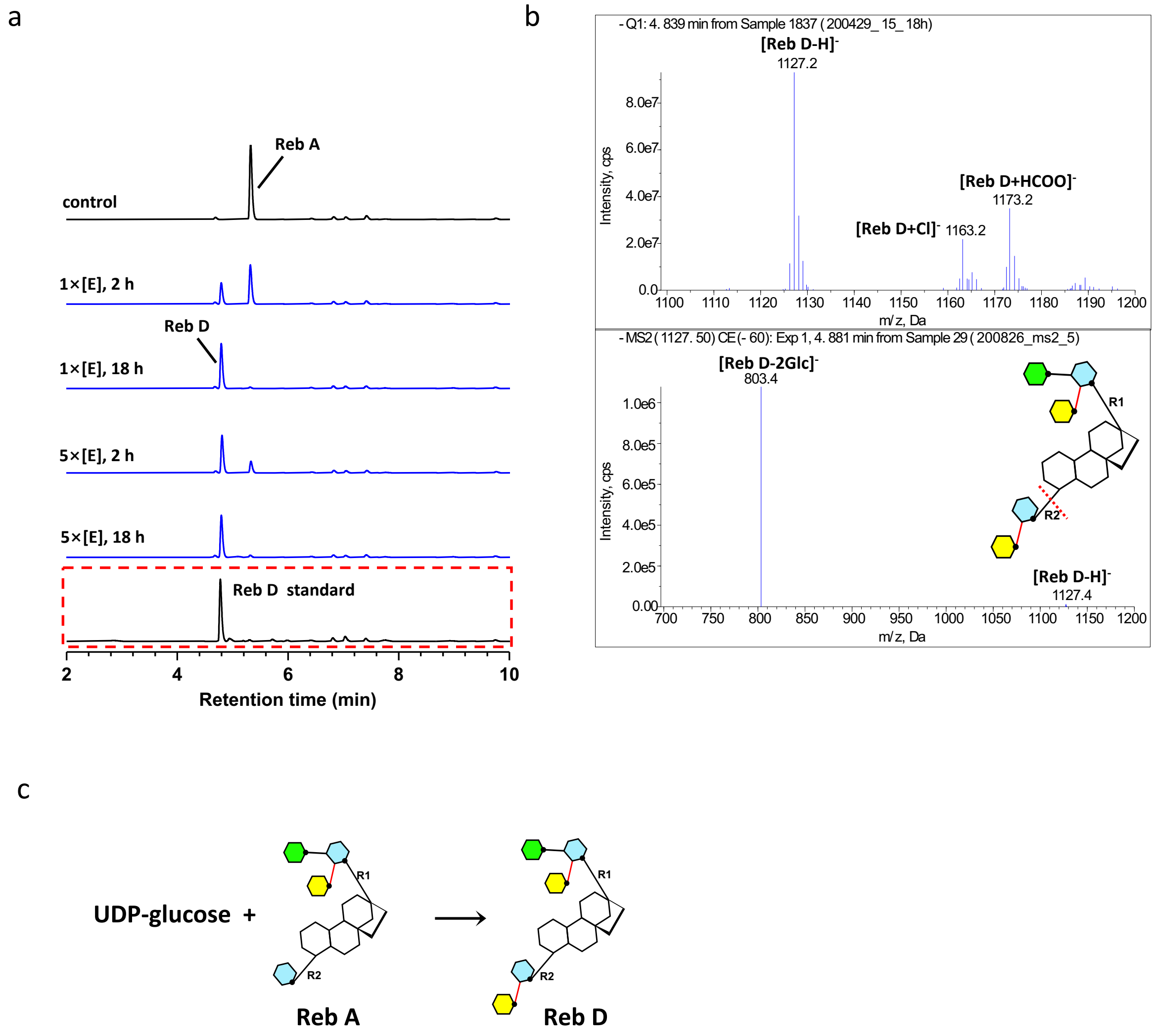

Supplementary Fig. 1 Reb A reaction with OsUGT91C1.

a LC-MS was used to monitor the reaction of Reb A with OsUGT91C1 and UDP-glucose. The HPLC traces record the 18 h control reaction without the enzyme (black); the incubations with the enzyme at 0.08 mg ml^{-1} (1x) for 2 h and 18 h (blue); the incubations were repeated at 0.4 mg ml^{-1} enzyme (5 fold increase in concentration, 5x). The authentic standard Reb D is shown in the red dashed box.

b Mass spectrometric analysis of the new peak in LC-MS was consistent with Reb D. The negative derived ions of Reb D are labeled in the upper panel. The negative parent ion [Reb D-H]$^-$ with m/z at 1127 was isolated and then characterized by MS/MS in the lower panel. The more labile ester bond breaks first in MS/MS, leading to the first mass loss of ester-linked glycan at the R2 end. The size of the mass loss indicates the number of sugars attached to the R2 end.

c Cartoon illustration of the reaction on Reb A that is catalyzed by OsUGT91C1.

Supplementary Table 1 Data collection and refinement statistics of X-ray structures

	Apo OsUGT91C1 (7ERY)	OsUGT91C1+UDP +Reb E (7ES0)	OsUGT91C1+UDP +ST (7ES1)	OsUGT91C1+UDP +STB (7ERX)	OsUGT91C1 H27A+UDP +Reb D (7ES2)
**Data collection**					
Space group	$P2_12_12_1$	$P2_12_12_1$	$P2_12_12_1$	$P2_12_12_1$	$P2_12_12_1$
Cell dimensions					
a, *b*, *c* (Å)	50.27, 79.84, 105.41	57.56, 83.08, 87.94	50.85, 83.10, 109.16	54.47, 83.15, 109.33	54.49, 83.53, 110.70
α, β, γ (°)	90, 90, 90	90, 90, 90	90, 90, 90	90, 90, 90	90, 90, 90
Resolution (Å)	50 - 1.77 (1.80-1.77) *	57.56 - 1.39 (1.43 - 1.39) *	46.09 - 1.66 (1.70 - 1.66) *	109.39 - 1.92 (1.97 - 1.92) *	36.88 – 2.34 (2.38 – 2.34)
R_{merge}	0.042(0.284)	0.037 (0.40)	0.083 (0.958)	0.055 (1.852)	0.056 (0.206)
$I / \sigma I$	35.1(4.7)	22.5 (1.3)	9.0(1.0)	14.5 (0.7)	15.8 (0.97)
Completeness (%)	100 (100)	69.4 (7.1)	99.9 (99)	99.7 (96.2)	100 (99.8)
Redundancy	6.5 (6.1)	5.8 (1.6)	5.9 (3.3)	6.2 (5.1)	6.0 (6.3)
CC (1/2)	0.99 (0.95)	0.998 (0.82)	0.996 (0.499)	0.999 (0.626)	0.977 (0.983)
**Refinement**					
Resolution (Å)	39.95 - 1.77	48.16 - 1.39	42.88 - 1.70	66.30 - 1.92	35.21 - 2.34
No. reflections	42099 (4135)	58628 (753)	55340 (5405)	38555 (1952)	21914 (2145)
R_{work} / R_{free}	0.166 / 0.198	0.159 / 0.174	0.179 / 0.204	0.188 / 0.214	0.196 / 0.243
No. atoms	3333	3769	3607	3286	3232
Protein	3142	3167	3151	3125	3099
Ligand/ion	-	119	59	76	103
Water	191	483	397	85	30
B-factors	33.0	18.0	32	52.0	63.2
Protein	22.8	14.8	16.3	52.2	62.6
Ligand/ion	-	21.9	31.4	64.1	84.5
Water	41.2	29.6	40.5	48.2	51.3
R.m.s. deviations					
Bond lengths (Å)	0.009	0.005	0.006	0.008	0.003
Bond angles (°)	1.54	1.36	1.44	1.48	1.28

*One crystal was used for data collection and structure determination. The resolution limits were determined by half-dataset correlation (CC (1/2)), and values in parentheses are for the highest-resolution shell.

Supplementary Fig. 2

a OsUGT91C1 + UDP + Reb E

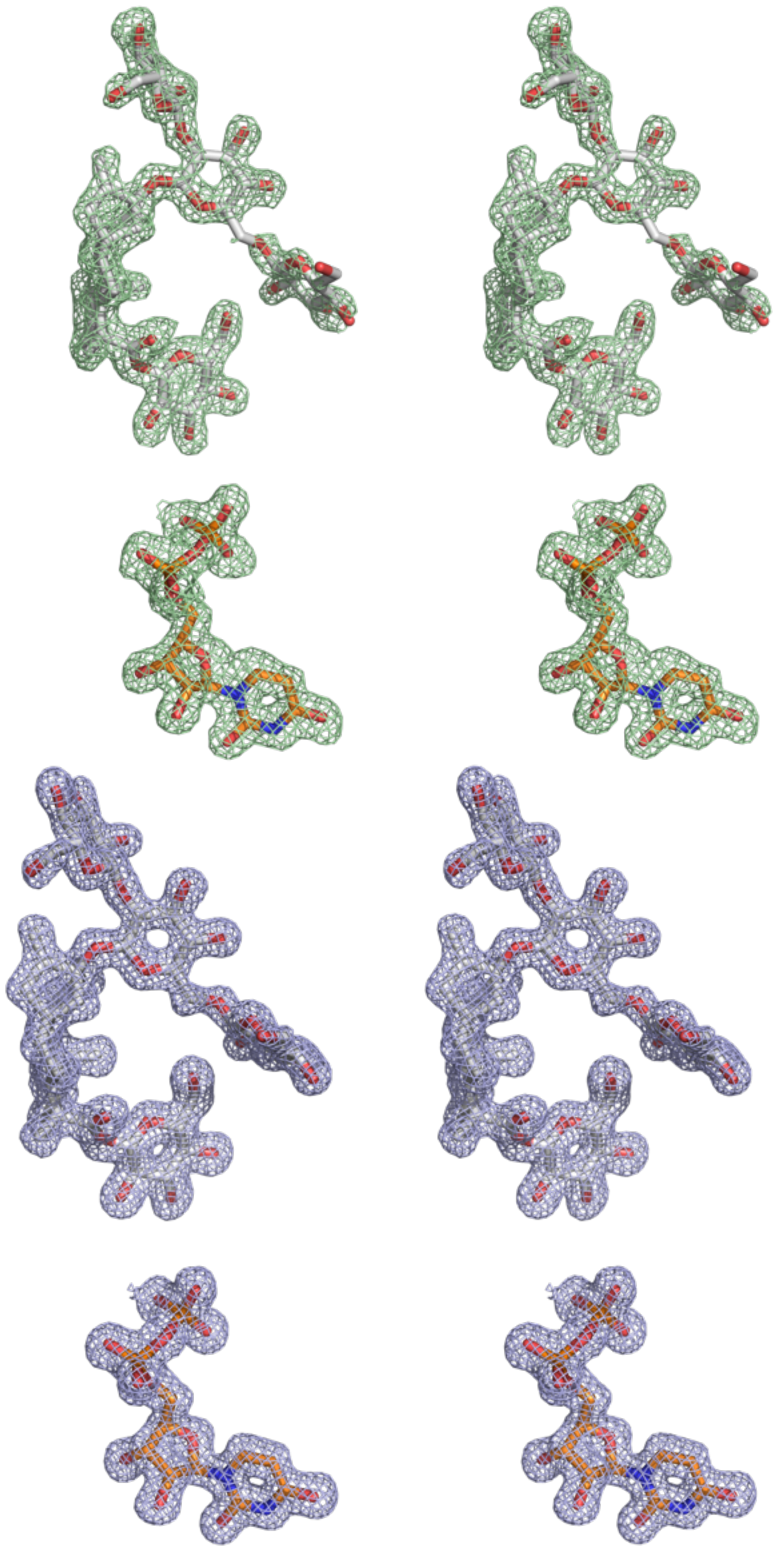

b OsUGT91C1 + UDP + ST

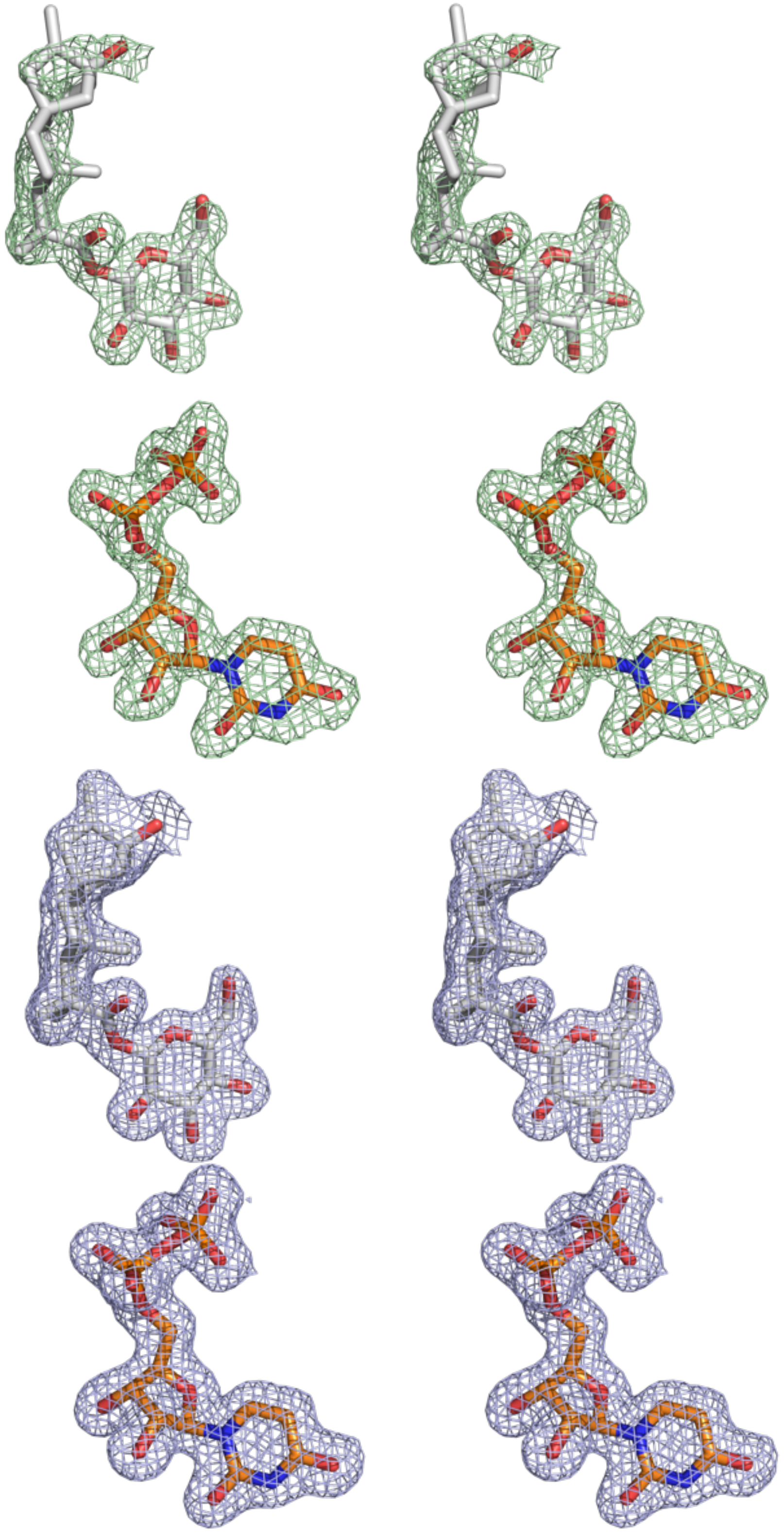

c OsUGT91C1 H27A + UDP + Reb D

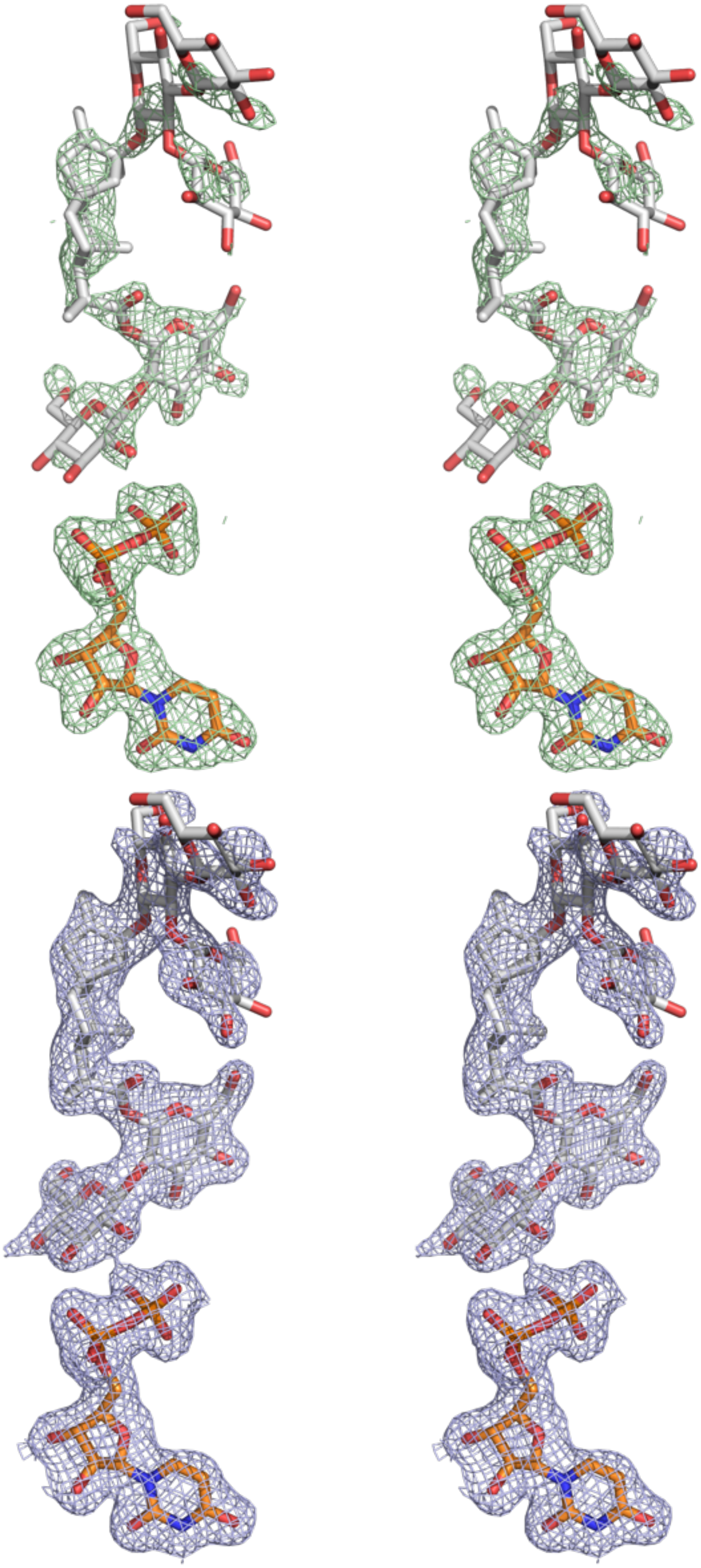

d OsUGT91C1 + UDP + STB

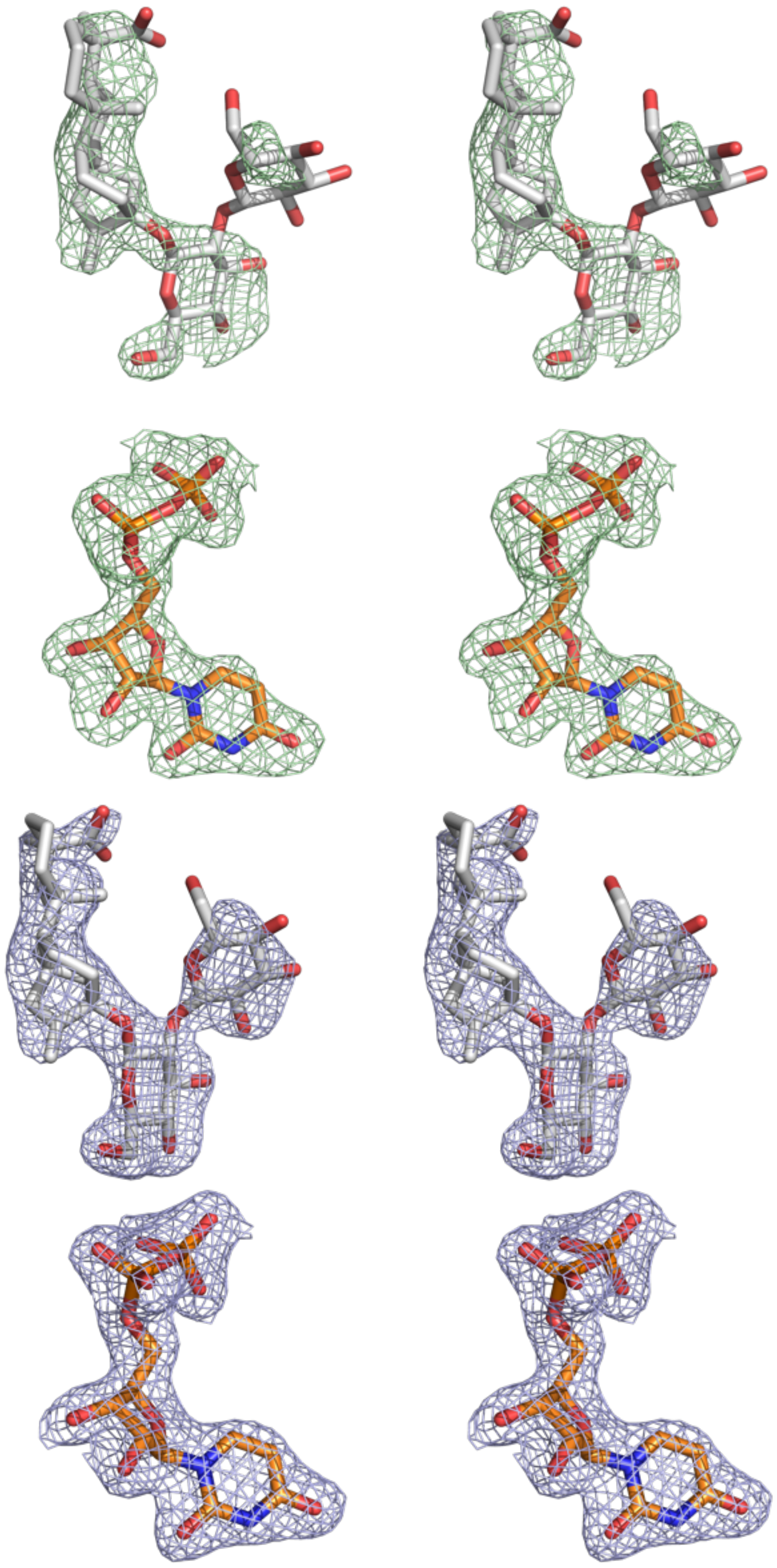

Supplementary Fig. 2 Experimental Fo-Fc difference electron density and the final refined 2Fo-Fc electron density for the bound ligands

Experimental Fo-Fc difference electron density contoured at 3σ in green and the final refined 2Fo-Fc electron density (1σ) in blue for the bound ligands in the structures. The final refined positions of the ligands are shown in stick with oxygen atoms red, nitrogen atoms blue, carbon atoms in the steviol compounds colored white, and in UDP orange.

a Ternary complex of OsUGT91C1/UDP/Reb E. Reb E was converted to a new steviol compound during crystallization.
b Ternary complex of OsUGT91C1/UDP/ST.
c Ternary complex of OsUGT91C1-H27A/UDP/Reb D.
d Ternary complex of OsUGT91C1/UDP/STB with the R1 end of STB at the active site

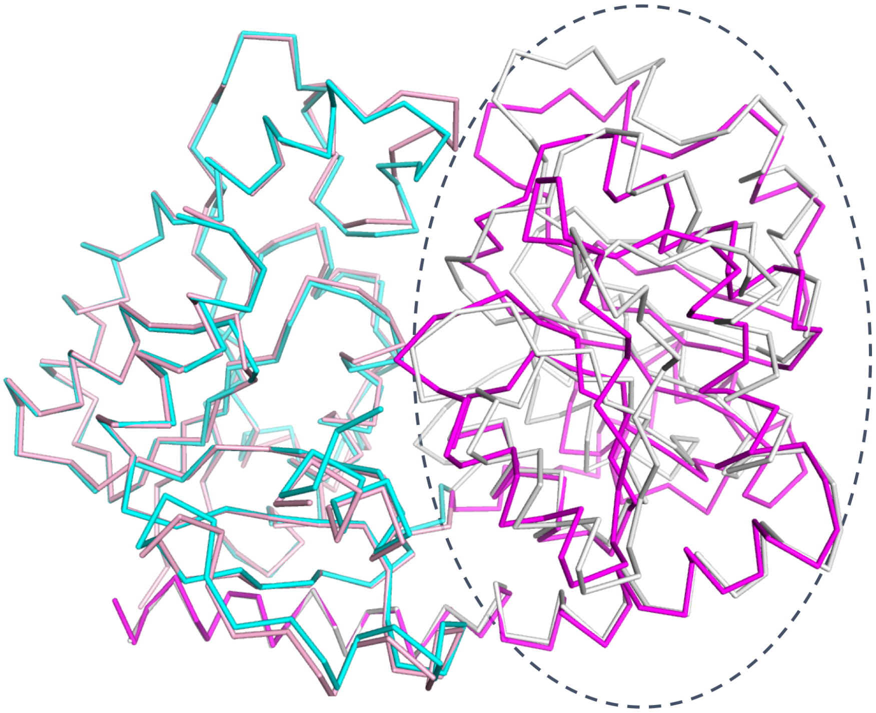

Supplementary Fig. 3 Conformational change upon complex formation.
Comparison of the backbone tracing between the apo and the structure in complex with UDP and Reb E. The N-terminus of the apo structure is shown in pink and the C-terminus shown in magenta, while the complex structure is in the same color scheme as Fig. 3 a. The C-terminal domain shifts upon UDP binding.

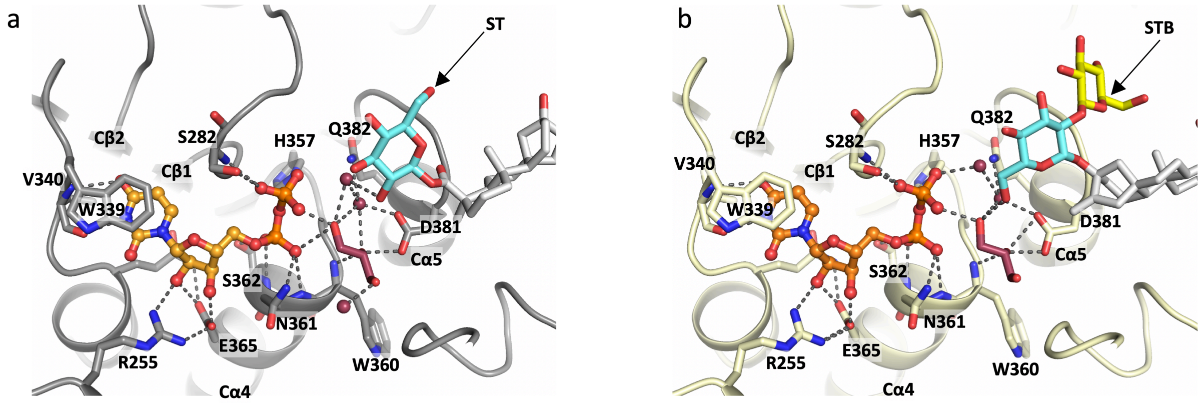

Supplementary Fig. 4 Identical UDP binding in all complexes of OsUGT91C1

The binding mode of UDP is identical regardless of which steviol compound is bound. In the ternary complex of OsUGT91C1/UDP/ST (**a**) and OsUGT91C1/UDP/STB (**b**), water molecules are shown as red spheres, and the glycerol is shown as sticks with carbon atoms colored raspberry. The water molecules and glycerol molecule occupy the volume where the glucose of UDP-glucose will bind.

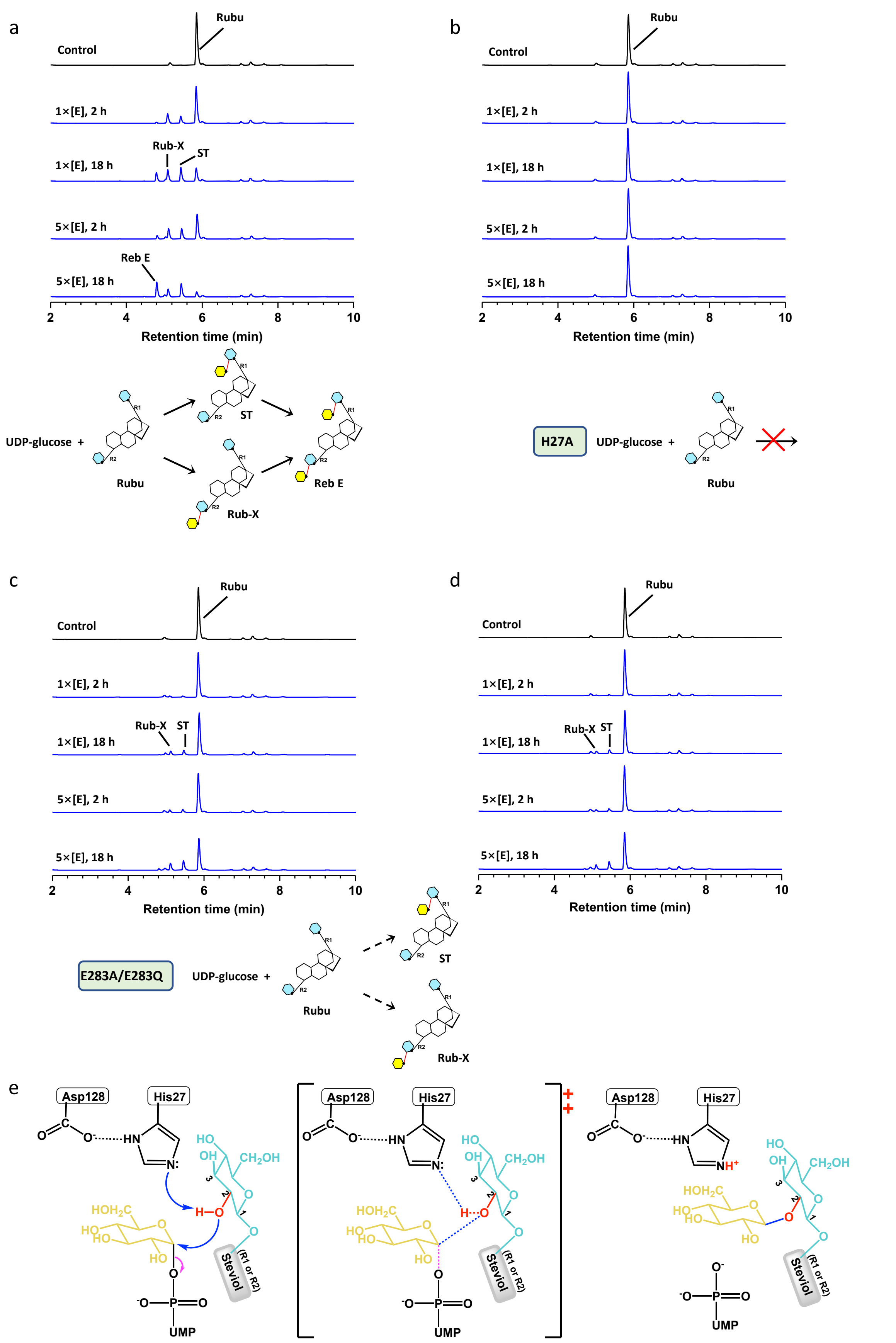

Supplementary Fig. 5 Role of His27 and Glu283 of OsUGT91C1 in β (1-2) sugar transfer

a-d LC-MS was used to monitor the reaction of Rubu with UDP-glucose and wild-type OsUGT91C1 (**a**), the mutants H27A (**b**), E283A (**c**), and E283Q (**d**). The HPLC traces record the

18 h control reaction without the enzyme (black); the incubations with the enzyme or mutants at 0.05 mg ml-1 (1x) for 2 h and 18 h (blue); the incubations were repeated with 0.25 mg ml-1 enzyme (5x). H27A mutant is inactive, and E283A and E283Q are catalytically impaired. The reaction schemes are shown for clarity. **e** S_N2 mechanism of OsUGT91C1 catalysis. His27, with the aid of Asp128, specifically deprotonates the 2-hydroxyl of glucose 1-R1 or 1-R2 and turns it to be a nucleophile, which attacks the sugar donor UDP-glucose. The predicted transition state of the S_N2 mechanism is shown in brackets.

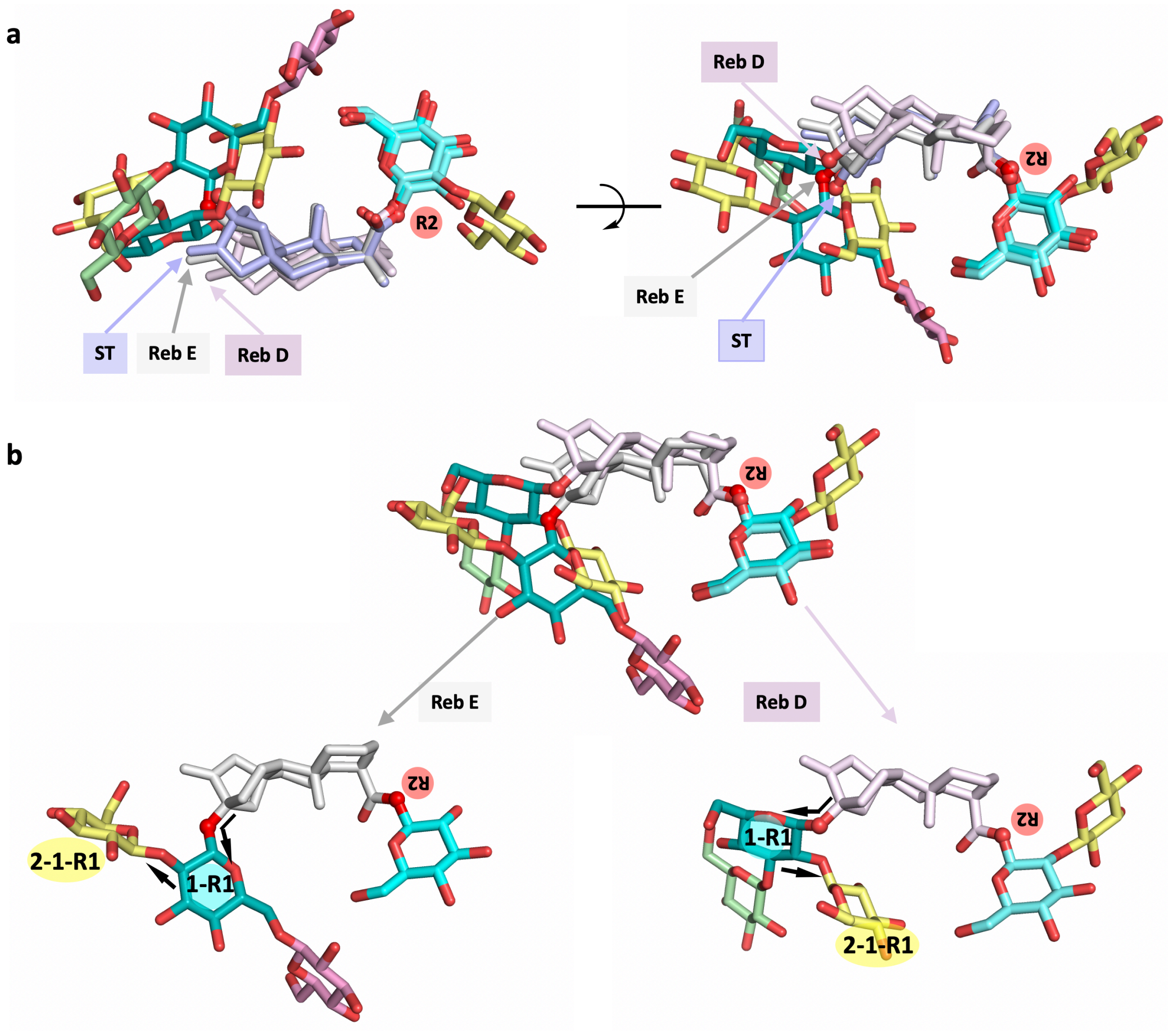

Supplementary Fig. 6 Comparison of the steviol compounds with the R2 end at the active site

a Positions of three steviol compounds, ST (steviol carbon atoms colored in light violet), Reb E (steviol carbon atoms colored in white), and Reb D (steviol carbon atoms colored in light pink) are shown. The oxygens of C19-carboxylate (R2) and C13-hydroxyl (R1) are shown as red sphere. The R2 ends occupy almost identical locations at the active site, while the R1 ends show significant variation at the "out" site.

b Comparison of two steviol compounds with tri-saccharide at the R1 end. The glycosidic bonds between glucose 1-R1 and the steviol aglycone in Reb E and Reb D adopt two different rotamers, resulting in a flip transition of glucose 1-R1 between two steviol compounds, Reb E and Reb D, and the different distribution of glucose 2-1-R1.

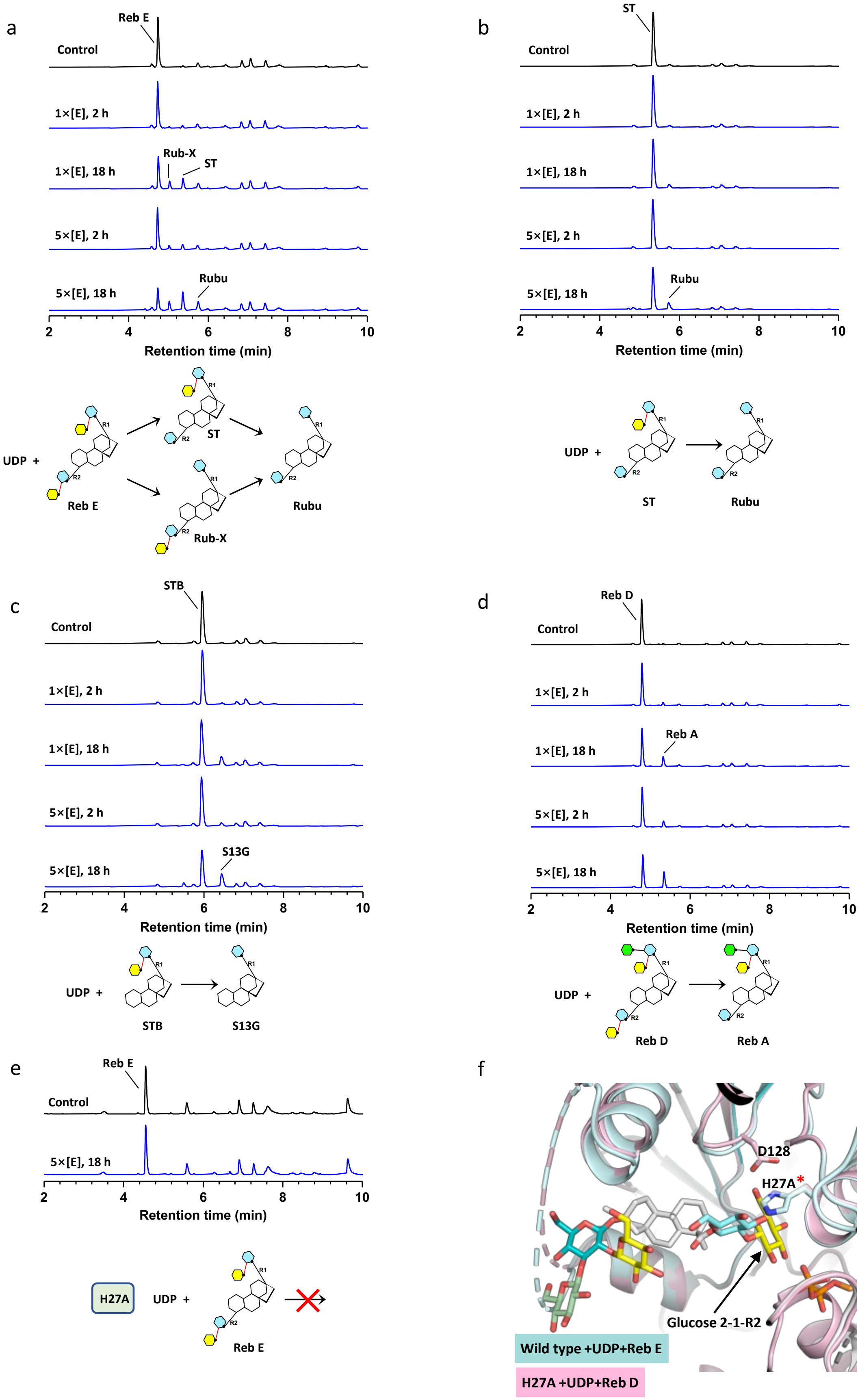

Supplementary Fig. 7 Cleavage of β (1-2) glycosidic bond by OsUGT91C1

a-d LC-MS analyses of the cleavage of β (1-2) glycosidic bond by OsUGT91C1 with the steviol compounds Reb E (**a**), ST (**b**), STB (**c**), and Reb D (**d**) in the presence of UDP. The HPLC traces

record the 18 h control reaction without the enzyme (black), the incubation with 0.30 mg ml^{-1} enzyme (1x) and UDP for 2 h and 18 h, repeated with 1.50 mg ml^{-1} enzyme (5x) (blue). OsUGT91C1 can cleave the glucose β (1-2) linkage at both the R1 and R2 ends when glucose 3-1-R1 or 3-1-R2 is absent.

e H27A mutation inactivates the enzymatic cleavage of β (1-2) glycosidic bond.

f Structure of H27A mutant in complex with UDP and Reb D. Reb D binds with the R2 end at the active site, and glucose 2-1-R2 is well-ordered in this inactive H27A mutant and occupies the space of previously filled by the imidazole side chain of His27, while glucose 2-1-R2 is absent in the structures of native OsUGT91C1 due to the enzyme-catalyzed cleavage.

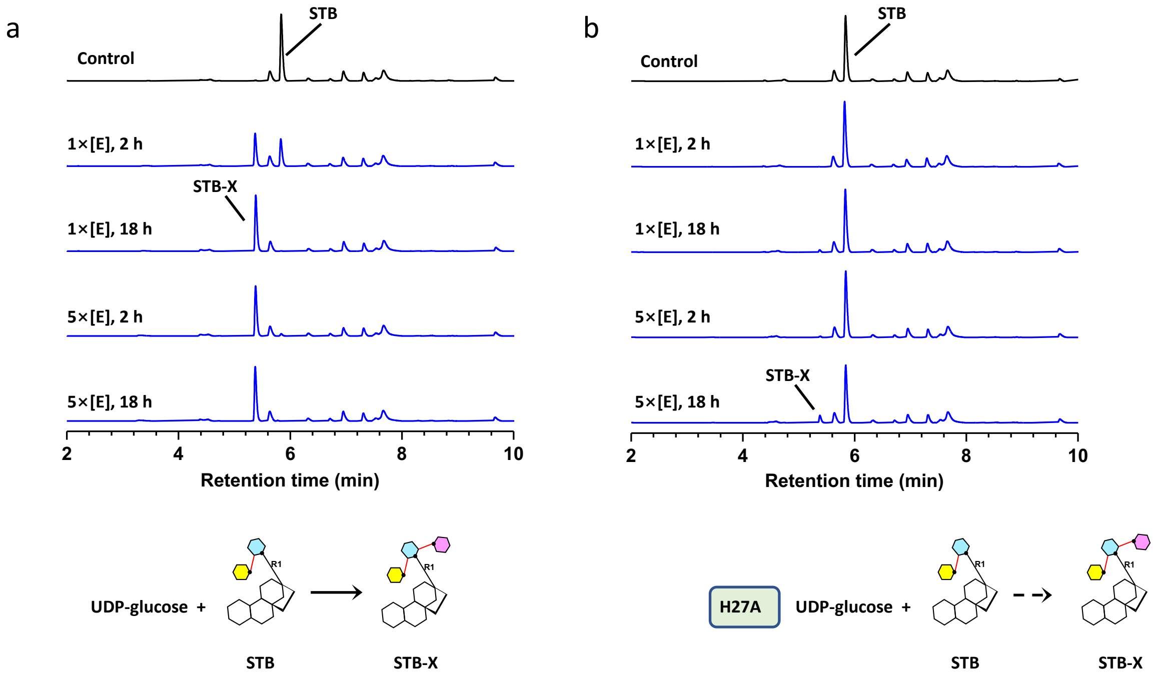

Supplementary Fig. 8 Role of His27 of OsUGT91C1 in β (1-6) sugar transfer.

a, b LC-MS analyses of the reaction of STB with UDP-glucose and wild-type OsUGT91C1 (**a**) and the mutants H27A (**b**). The HPLC traces record the 18 h control reaction without the enzyme (black), the incubation of the wild type enzyme at 0.15 mg ml^{-1} (1x) for 2 h and 18 h, repeated with 0.75 mg ml^{-1} enzyme (5x) (blue). The wild-type enzyme showed β (1-6) enzymatical glucosylation on STB; the H27A mutant is almost inactive. Cartoon of the reaction catalyzed by the enzyme is shown.

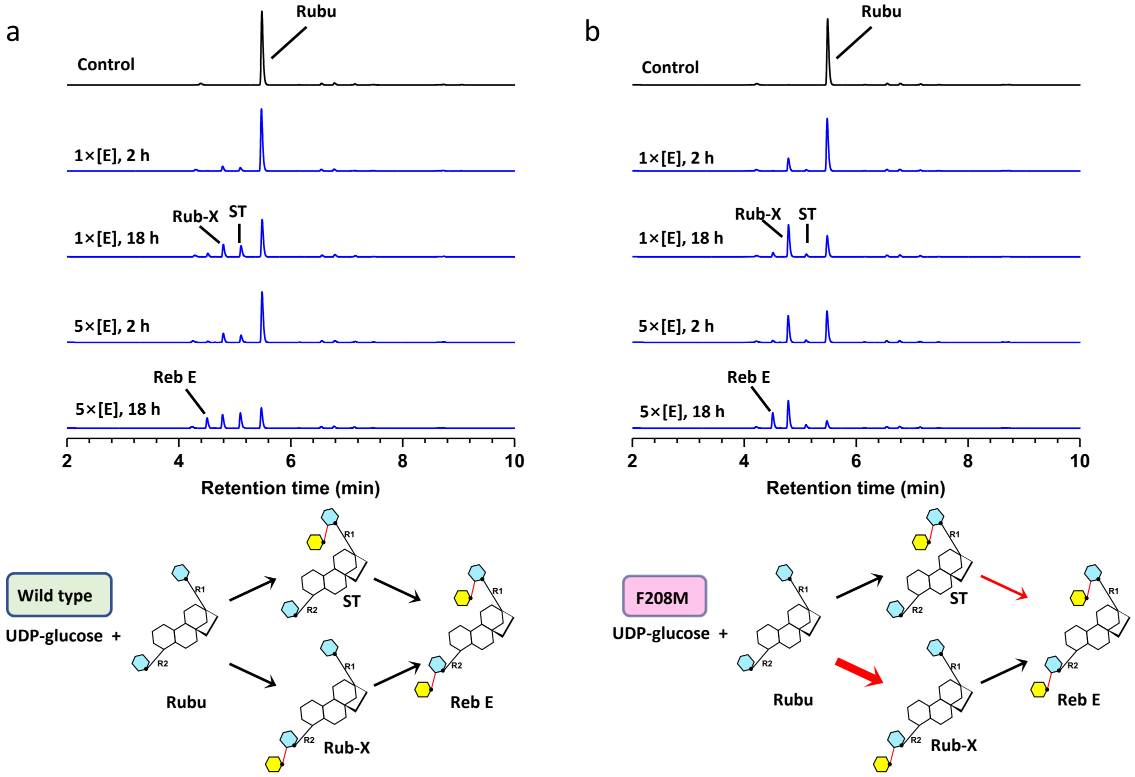

Supplementary Fig. 9 Engineering to enhance β (1-2) sugar transfer.
a-b LC-MS analyses of the reaction of Rubu with UDP-glucose and the wild-type OsUGT91C1 (**a**) and the mutant F208M (**b**). The HPLC traces record the 18 h control reaction without the enzyme (black), the reaction with 0.05 mg ml^{-1} enzyme (1x) for 2 h and 18 h, and repeated with 0.25 mg ml^{-1} enzyme (5x) (blue). The product yields of the mutant F208M differed from the wild type and demonstrated a preference for R2 addition. A cartoon illustrates the change in the R1 vs R2 preference.

Supplementary Figure 10

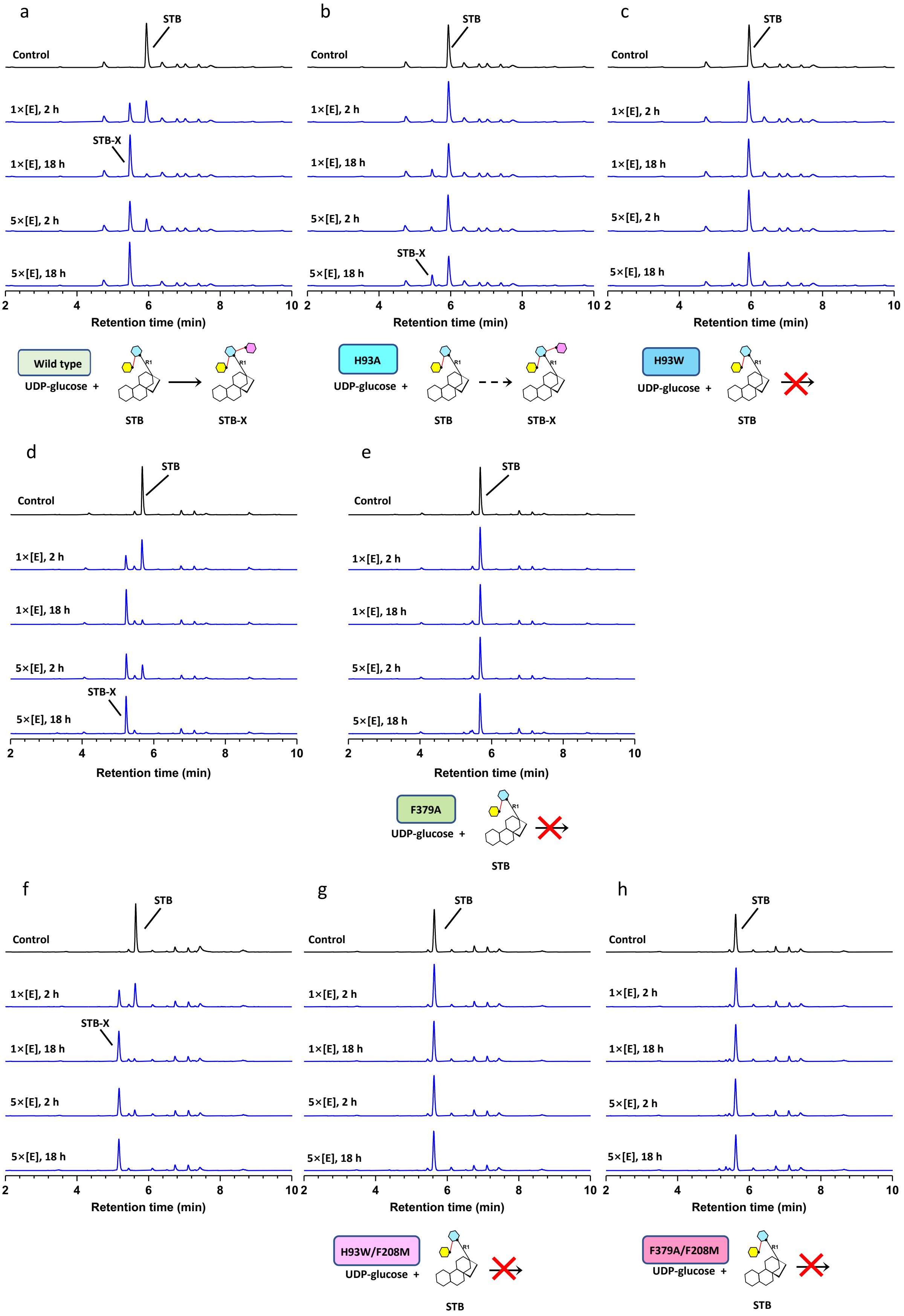

Supplementary Figure 10 Engineering to suppress the unwanted β (1-6) sugar transfer.

a-h LC-MS analyses of the reaction of STB with three individual groups of the wild type and the mutants. Group 1, the wild type (**a**), H93A (**b**), and H93W (**c**); group 2, the wild type (**d**) and F379A (**e**); and group 3, the wild type (**f**), H93W/F208M (**g**) and F379A/F208M (**h**). The HPLC traces record the 18 h control reaction without the enzyme (black), the reaction with 0.15 mg ml^{-1} enzyme (1x) for 2 h and 18 h, and the repeated reaction with 0.75 mg ml^{-1} enzyme (5x) for 2 h and 18 h (blue). Cartoons of the reaction illustrate the suppression of β (1-6) sugar transfer.

Supplementary Table 2 Synthetic encoding sequence of OsUGT91C1, the primers, and the reagents used in this study

The synthetic OsUGT91C1 encoding sequence	ATGGATAGCGGTTATAGTAGCAGTTATGCCGCAGCCGCCGGCATGCATGTTGTGATTTGCCCGT<br>GGCTGGCCTTTGGTCATCTGCTGCCGTGCTTAGACCTGGCCCAGCGTCTGGCCAGCCGTGGTC<br>ACCGTGTTAGCTTTGTGAGCACCCCGCGTAATATCAGCCGTCTGCCGCCGGTTCGTCCGGCATT<br>AGCCCCGCTGGTGGCATTTGTGGCCTTACCGCTGCCGCGTGTTGAGGGTCTGCCTGATGGCGC<br>CGAAAGTACCAACGACGTGCCGCATGACCGCCCGGATATGGTGGAGCTGCATCGTCGCGCCTT<br>TGATGGTCTGGCAGCCCCGTTTAGCGAGTTTCTGGGCACAGCCTGCGCCGATTGGGTGATCGT<br>TGACGTGTTTCATCACTGGGCAGCCGCAGCCGCCCTGGAACATAAAGTTCCGTGCGCAATGAT<br>GCTGCTGGGTAGCGCCCACATGATTGCCAGCATTGCCGATCGTCGCCTGGAACGCGCAGAGA<br>CCGAAAGCCCGGCAGCAGCAGGTCAAGGTCGTCCTGCCGCAGCCCCGACCTTTGAAGTGGC<br>CCGCATGAAACTGATCCGTACCAAAGGTAGTAGCGGCATGAGCCTGGCCGAACGCTTTAGCCT<br>GACCCTGAGCCGCAGTAGCCTGGTGGTTGGTCGCAGTTGTGTGGAATTCGAGCCGGAAACAG<br>TGCCGCTGCTGAGCACCCTGCGCGGCAAACCGATCACCTTTCTGGGCCTGATGCCGCCGTTAC<br>ATGAAGGCCGTCGTGAAGATGGTGAAGATGCCACAGTGCGTTGGCTGGATGCACAGCCGGCC<br>AAAAGCGTTGTGTACGTTGCCCTGGGTAGCGAAGTTCCTCTGGGTGTGGAAAAGGTGCACGA<br>ACTGGCACTGGGTCTGGAACTGGCCGGTACCCGCTTCCTGTGGGCCTTACGTAAACCTACCGG<br>TGTTAGCGATGCCGATCTGCTGCCGGCAGGTTTTGAGGAACGTACCCGTGGTCGCGGTGTTGT<br>GGCAACACGCTGGGTTCCGCAGATGAGCATTCTGGCCCATGCCGCCGTGGGTGCCTTTCTGA<br>CCCATTGTGGCTGGAATAGCACCATCGAAGGCCTGATGTTCGGCCATCCTCTGATCATGCTGCC<br>TATCTTCGGTGATCAGGGTCCGAACGCACGCCTGATTGAAGCAAAGAATGCCGGTCTGCAGGT<br>GGCACGTAACGATGGCGACGGTAGCTTCGATCGTGAAGGCGTTGCCGCCGCAATTCGCGCCG<br>TTGCAGTTGAAGAAGAGAGCAGCAAGGTGTTCCAGGCCAAAGCCAAAAAACTGCAGGAGAT<br>CGTGGCCGATATGGCATGCCATGAGCGCTACATCGATGGCTTCATCCAGCAGCTGCGCAGCTAT<br>AAAGATCTCGAGCACCACCACCACCACCAC
Primers used to subclone OsUGT91C1 into pET28b	5’ -GAAGGAGATATACCATGGATGGATAGCGGTTATAGTAGCA-3’<br>5’ -TTGTCGACGGAGCTCGAATTCTCAGTGGTGGTGGTGGTG-3’
Primers used for the mutations	
H27A	5’ -CGTGGCTGGCCTTTGGTGCTCTGCTGCCGTGCTTAG-3’<br>5’ -TAAGCACGGCAGCAGAGCACCAAAGGCCAGCCACGG-3’
E283A	5’ -GTTGCCCTGGGTAGCGCAGTTCCTCTGGGTGTG-3’<br>5’ -CACACCCAGAGGAACTGCGCTACCCAGGGCAAC-3’
E283Q	5’ -GTTGCCCTGGGTAGCCAAGTTCCTCTGGGTGTG-3’<br>5’ -CACACCCAGAGGAACTTGGCTACCCAGGGCAA-3’
H93A	5’ -ACCAACGACGTGCCGGCTGACCGCCCGGATATG-3’<br>5’ -CATATCCGGGCGGTCAGCCGGCACGTCGTTGGT-3’
H93W	5’ -AAAGTACCAACGACGTGCCGTGGGACCGCCCGGATATGGTGGA-3’<br>5’ -TCCACCATATCCGGGCGGTCCCACGGCACGTCGTTGGTACTTT-3’
F208M	5’ -TGAGCCTGGCCGAACGCATGAGCCTGACCCTGAGC-3’<br>5’ -GCTCAGGGTCAGGCTCATGCGTTCGGCCAGGCTCA-3’
F379A	5’ -CTGATCATGCTGCCTATCGCCGGTGATCAGGGTCCGAACG-3’<br>5’ -CGTTCGGACCCTGATCACCGGCGATAGGCAGCATGATCAGA-3’
Reagents used in this study	
UDPG	U4625/Sigma-Aldrich
UDP	94330/Sigma-Aldrich
RUBUSOSIDE	ASB-00018412/ChromaDex
STEVIOSIDE	ASB-00019351/ChromaDex
REBAUDIOSIDE A	ASB-00018226/ChromaDex
REBAUDIOSIDE D	ASB-00018229/ChromaDex

REBAUDIOSIDE E	ASB-00018235/ChromaDex
STEVIOLBIOSIDE	ASB-00019349/ChromaDex
Steviol-13-O-monoglucoside (S13G)	Manufactured on customer request/EnzymeWorks, China
UDP-Glo™ Glycosyltransferase Assay	V6963/Promega
