# Supplementary material for: Catalytic flexibility of rice glycosyltransferase OsUGT91C1 for the production of palatable steviol glycosides
Source: Nat Commun. 2021 Dec 2;12:7030. doi: 10.1038/s41467-021-27144-4 (PMC8639739; doi:10.1038/s41467-021-27144-4)
Supplement: Supplementary file 3 — Reporting Summary [file 41467_2021_27144_MOESM3_ESM.pdf]

nature portfolio

Corresponding author(s): Xiaofeng Zhu

Last updated by author(s): Oct 25, 2021

Reporting Summary

Nature Portfolio wishes to improve the reproducibility of the work that we publish. This form provides structure for consistency and transparency in reporting. For further information on Nature Portfolio policies, see our Editorial Policies and the Editorial Policy Checklist.

Statistics

For all statistical analyses, confirm that the following items are present in the figure legend, table legend, main text, or Methods section.

n/a	Confirmed	
☒	☐	The exact sample size (*n*) for each experimental group/condition, given as a discrete number and unit of measurement
☐	☒	A statement on whether measurements were taken from distinct samples or whether the same sample was measured repeatedly
☒	☐	The statistical test(s) used AND whether they are one- or two-sided<br>*Only common tests should be described solely by name; describe more complex techniques in the Methods section.*
☒	☐	A description of all covariates tested
☒	☐	A description of any assumptions or corrections, such as tests of normality and adjustment for multiple comparisons
☒	☐	A full description of the statistical parameters including central tendency (e.g. means) or other basic estimates (e.g. regression coefficient) AND variation (e.g. standard deviation) or associated estimates of uncertainty (e.g. confidence intervals)
☒	☐	For null hypothesis testing, the test statistic (e.g. *F*, *t*, *r*) with confidence intervals, effect sizes, degrees of freedom and *P* value noted<br>*Give P values as exact values whenever suitable.*
☒	☐	For Bayesian analysis, information on the choice of priors and Markov chain Monte Carlo settings
☒	☐	For hierarchical and complex designs, identification of the appropriate level for tests and full reporting of outcomes
☒	☐	Estimates of effect sizes (e.g. Cohen's *d*, Pearson's *r*), indicating how they were calculated

Our web collection on statistics for biologists contains articles on many of the points above.

Software and code

Policy information about availability of computer code

Data collection	LC-MS data were collected using Analyst 1.6.2 software (AB SCIEX, USA), Varioskan™ Flash multimode microplate reader used in the enzyme assays.
Data analysis	LC-MS data were analyzed using Analyst 1.6.2 software (AB SCIEX, USA).<br>X-ray data were analyzed using DIALS (version 3.0) in CCP4i2 package (version 1.0), CRANK2 (version 2.0), Phaser (version 2.8), Coot (version 0.8), Refmac5 (version 5.8), ProDrg (version 2.5), Acedrg (version 217) in CCP4i package (version 7.1) and Privateer42 (version MKIII) in CCP4i2 package (version 1.1).<br>Skanlt software 2.4.3 RE for Varioskan Flash controlled the microplate reader and recorded the data.

For manuscripts utilizing custom algorithms or software that are central to the research but not yet described in published literature, software must be made available to editors and reviewers. We strongly encourage code deposition in a community repository (e.g. GitHub). See the Nature Portfolio guidelines for submitting code & software for further information.

Data

Policy information about availability of data

All manuscripts must include a data availability statement. This statement should provide the following information, where applicable:

- Accession codes, unique identifiers, or web links for publicly available datasets
- A description of any restrictions on data availability
- For clinical datasets or third party data, please ensure that the statement adheres to our policy

The coordinates and structure factors have been deposited in the Protein Data Bank under accession codes 7ERY, 7ES0, 7ES1, 7ERX, 7ES2. The source data underlying the kinetic parameters in Table 1 are provided with this paper as a Source Data file. All relevant data generated in this study are provided in the main

text, the Supplementary information, or Source data file. Four glycosyltransferases (UGTs) of S. rebaudiana are annotated as GT1 family members in the Carbohydrate-Active enZymes (CAZy) Database by searching their GenBank accession codes: AAR06916.1, AAR06920.1, ACE87855.1, and AGL95113.1.

Field-specific reporting

Please select the one below that is the best fit for your research. If you are not sure, read the appropriate sections before making your selection.

☒ Life sciences ☐ Behavioural & social sciences ☐ Ecological, evolutionary & environmental sciences

For a reference copy of the document with all sections, see nature.com/documents/nr-reporting-summary-flat.pdf

Life sciences study design

All studies must disclose on these points even when the disclosure is negative.

Sample size	1) No statistical methods were used to predetermine sample sizes for the biochemical assays. Required experimental sample sizes were chosen according to common practice in enzymology (three independent experiments) and including appropriate positive and negative controls. LC-MS samples were prepared and measured in technical triplicate. The data of LC-MS and LC-MS/MS identified the product consistently, and showed the same profile of substrate consumption and product production. The enzymatic kinetic data were performed in technical triplicate (n=3).
Data exclusions	No data were excluded.
Replication	All experimental findings were confirmed with at least three independent replicates. All attempts at replication succeeded in showing the same conclusion. The enzymatic kinetic data were performed in technical triplicate (n=3) and show good fit to the Michaelis–Menten equation. The original data and data-fitting were included in Source Data file.
Randomization	Randomization is not relevant to the majority of in vitro experiments of this study, because the experiments in this manuscript were performed with purified individual enzyme samples, which are essentially identical in crystallization experiments and enzyme assays. Randomization was used to assign 5% crystal diffraction data with the R-free flags during the structure refinement to avoid over-fitting. We kept using the same set of R-free flags for the other datasets, which have the same space group and roughly the same cell dimensions, which is the good practice in processing datasets associated with protein-ligand complex structures.
Blinding	Blinding was not performed in this study as the results do not require subjective interpretation.

Reporting for specific materials, systems and methods

We require information from authors about some types of materials, experimental systems and methods used in many studies. Here, indicate whether each material, system or method listed is relevant to your study. If you are not sure if a list item applies to your research, read the appropriate section before selecting a response.

Materials & experimental systems

n/a	Involved in the study	
☒	☐	Antibodies
☒	☐	Eukaryotic cell lines
☒	☐	Palaeontology and archaeology
☒	☐	Animals and other organisms
☒	☐	Human research participants
☒	☐	Clinical data
☒	☐	Dual use research of concern

Methods

n/a	Involved in the study	
☒	☐	ChIP-seq
☒	☐	Flow cytometry
☒	☐	MRI-based neuroimaging
